# Risk Factors Influencing Seeking Emergency Medical Service in Urban and Rural China Among Participants With a Previous Transient Ischemic Attack

**DOI:** 10.3389/fneur.2020.620157

**Published:** 2021-02-04

**Authors:** Jing Yuan, Guang-Liang Shan, Sheng-De Li, Chun-Peng Gao, Li-Ying Cui, Bin Peng

**Affiliations:** ^1^Department of Neurology, Peking Union Medical College Hospital, Peking Union Medical College, Chinese Academy of Medical Sciences, Beijing, China; ^2^Department of Epidemiology and Statistics, Institute of Basic Medical Sciences, Chinese Academy of Medical Sciences, School of Basic Medicine, Peking Union Medical College, Beijing, China; ^3^Disease Control and Prevention Office, Dalian Municipal Central Hospital, Dalian, China

**Keywords:** transient ischemic attack, stroke, cerebrovascular disease, risk factors, emergency medical service, urban, rural, China

## Abstract

**Objective:** It is critical to identify factors that significantly impede the correct action of calling emergency medical service (EMS) in the high-risk population with a previous history of transient ischemic attack (TIA) and further explore the urban–rural difference in China.

**Methods:** Participants with previous TIA from the China National Stroke Screening Survey and its branch study (FAST-RIGHT) were interviewed cross-sectionally (*n* = 2,036). The associations between the outcome measure of not calling EMS and multiple potential risk factors were examined, including demographic information, live (or not) with families, medical insurance type, urban or rural residence, awareness of stroke symptoms, annual personal income, presence of cardiovascular disease or risk factors, and stroke history in family members or friends. The sample was further stratified to explore the urban–rural difference by their residency.

**Results:** The proportion of not calling EMS was 36.8% among all participants with previous TIA, and these were 21.7 and 48.4% among urban and rural participants, respectively. Among rural participants, risk factors that were significantly associated with not calling EMS included primary school education [odds ratio (OR) 2.50, 95% confidence interval (CI) 1.89–3.33], living with family (OR 2.09, 95% CI 1.33–3.36), unaware stroke symptoms (OR 2.60, 95% CI 1.81–3.78), and low income (OR 1.57, 95% CI 1.19–2.07). Among urban participants, only low income was significantly associated with an increased risk of not calling EMS (OR 1.74, 95% CI 1.10–2.72).

**Conclusions:** Rural residents with previous TIA in China had a higher percentage of not calling EMS. Multiple risk factors have been identified that call for targeted intervention strategies.

## Introduction

Patients with a previous transient ischemic attack (TIA) have a higher risk for incident stroke. The stroke incidence after the TIA episode is 7.27% within 12 months (1). In China, the age-standardized TIA prevalence was 2.27%, and it was estimated that 23.9 million people in China might have experienced a TIA (2). Disproportionately, the vast majority (86%) of them did not seek medical attention and, therefore, did not receive appropriate care (2). An earlier hospital-based survey in 2007–2008 found that 64.78% of patients with TIA had delayed presentation to the hospital (>24 h) (3).

The correct action of calling an emergency medical service (EMS) is a crucial step to shorten the prehospital delay (3, 4). Acknowledgedly, active countermeasures have been taken in China in recent years. The China National Stroke Screening Survey (CNSSS) was established in 2013 as large-scale community-based stroke surveillance and educational program among residents of all 31 provinces in China (not including Hong Kong, Macau, or Taiwan) (5, 6). An educational program called “Stroke 120” was also launched in 2016 to improve public awareness, which has witnessed a good effect (7, 8). Nonetheless, based on the results of the FAST-RIGHT study, which is a branch survey of the CNSSS aiming to investigate stroke awareness, the rates of unawareness and not calling EMS were still 18.1% and 39.1%, respectively, in the general population (9). This result indicates that stroke awareness does not necessarily translate into appropriate actions.

Multiple factors have been reported to influence the seeking of EMS in the general population (9). However, it is critical to identify factors that significantly impede the correct action in the high-risk population with a previous TIA.

More importantly, Wang et al. (10) reported that China's stroke burden has increased over the past 30 years and remains particularly high in rural areas. The prevalence, incidence, and mortality rates of stroke were significantly higher in rural areas than in urban areas. In China today, high economic growth and high urban–rural income disparities coexist (11, 12). Although China has seen a dramatic decline in poverty (13, 14), according to the National Bureau of Statistics reports, there were still 5.51 million people in 2019 whose annual income was below the current poverty standard in rural areas (14, 15). Besides the imbalanced economic development, the urban–rural difference is also reflected in other aspects, such as the prevalence of vascular risk factors (16–20), access to healthcare (21), healthcare utilization (22), etc.

Therefore, this paper investigates the factors that influence the intention of seeking EMS in China among the high-risk population with previous TIA, and further explore the urban–rural difference. This will contribute to formulate pertinent countermeasures to the targeted population and to alleviate China's social and economic burden.

## Materials and Methods

### Study Design and Participants

The FAST-RIGHT study is a cross-sectional branch survey of the CNSSS (details in previous publications) (9, 23, 24). Briefly, in the CNSSS, residents aged 40 years and older were sampled from 221 counties in 31 provinces of mainland China using a two-stage stratified sampling framework. Among the 221 counties, a subsample of residents (*n* = 187,723) being interviewed in person between January 1 and May 31, 2017, from 69 counties covering most provinces constituted the FAST-RIGHT study [see the online-only data supplement by Li et al. (9)]. FAST-RIGHT participants with previous TIA were eligible to be included in the present analysis (*n* = 2,036). TIA diagnosis was inquired and determined by the study physicians based on medical history, signs, and imaging results (CT or MRI). The Peking Union Medical College Hospital Institutional Review Board approved the study's protocols. Written informed consent was obtained from all participants.

### Data Collection and Assessment of Risk Factors and Intention to Call Emergency Medical Service

Participants were interviewed face-to-face by trained staff using standard questionnaires designed by the study team (9, 23). We collected factors of demographic information (age, sex, and education), medical insurance type, urban or rural residency, awareness of stroke symptoms, annual personal income (< $731 as low income), and the yes-or-no questions of living with family, stroke occurrence in family members or friends, and the presence of cardiovascular disease or risk factors (defined as having one of the following conditions, including hypertension, diabetes, hyperlipidemia, heart disease, smoking, or drinking alcohol). The outcome of interest is the intention of calling EMS (yes or no). Participants were asked how they would act if they had suspected stroke symptoms from the three choices of home observing, waiting for family members to go to a hospital, or calling an ambulance immediately. The final option was regarded as the correct action.

### Statistical Analysis

Count and percentage presented characteristics distribution for all participants with previous TIA, two groups by intention to call (or not) EMS (Table 1) and by urban or rural residency (Table 2). The chi-square test was used for group comparison. Multivariate logistic regression was performed to examine the association between the binary dependent variable of intention to call EMS (the probability of not calling EMS was modeled) and potential risk factors. The independent variables were categorical and entered into the models based on our previous analysis (9) and clinically significant judgment. The models were performed in the total sample of all TIA participants and the subsamples by urban–rural residency. *P*-value was set to <0.05, and SAS version 9.3 (Statistical Analysis System, RRID:SCR_008567) was used for analysis.

**Table 1:** Characteristics of all participants with previous TIA history and by intention to call EMS.

	**All TIA, n (%)**	**Not call EMS, n (%)**	**Call EMS, n (%)**	***P***
*N*	2,036	749	1,287	
Age, ≥ 65 years	1,090 (53.5)	409 (54.6)	681 (52.9)	0.46
Sex, male	809 (39.7)	302 (40.3)	507 (39.4)	0.68
Education				**<0.0001**
middle school above	990 (48.6)	254 (33.9)	736 (57.2)	
primary school	1,046 (51.4)	495 (66.1)	551 (42.8)	
Medical insurance, rural	960 (47.7)	459 (61.6)	501 (39.5)	**<0.0001**
Stroke in acquaintance, yes	708 (34.8)	215 (28.7)	493 (38.3)	**<0.0001**
Present CVD or risk factors	1,430 (70.2)	492 (65.7)	938 (72.9)	**0.0006**
Income status, income < $731	889 (43.7)	445 (59.6)	444 (34.5)	**<0.0001**
Living status, with family	1,888 (92.9)	702 (94.1)	1,186 (92.2)	0.11
Awareness, unaware	272 (13.4)	151 (20.2)	121 (9.4)	**<0.0001**
Site, rural	1,154 (56.7)	558 (74.5)	596 (46.3)	**<0.0001**

*CVD, cardiovascular disease. Statistical significance (P <0.05) was indicated in bold*.

**Table 2:** Urban–rural difference in characteristics distribution among all TIA participants and by intention to call EMS.

	**All TIA, n (%)**			**Not call EMS, n (%)**			**Call EMS, n (%)**		
	**Urban *n =* 882**	**Rural *n =* 1,154**	***P***	**Urban *n =* 191**	**Rural *n =* 558**	***P***	**Urban *n =* 691**	**Rural *n =* 596**	***P***
Age, ≥ 65 years	480 (54.4)	610 (52.9)	0.48	111 (58.1)	298 (53.4)	0.26	369 (53.4)	312 (52.4)	0.71
Sex, male	348 (39.5)	461 (40.0)	0.82	88 (46.1)	214 (38.4)	0.06	260 (37.6)	247 (41.4)	0.16
Education			**<0.0001**			**<0.0001**			**<0.0001**
primary school	278 (31.5)	768 (66.6)		63 (33.0)	432 (77.4)		215 (31.1)	336 (56.4)	
middle school above	604 (68.5)	386 (33.5)		128 (67.0)	126 (22.6)		476 (68.9)	260 (43.6)	
Medical insurance, rural	60 (7.0)	900 (78.1)	**<0.0001**	20 (10.6)	439 (79.0)	**<0.0001**	40 (6.0)	461 (77.4)	**<0.0001**
Stroke in acquaintance, yes	395 (44.8)	313 (27.1)	**<0.0001**	70 (36.7)	145 (26.0)	**0.005**	325 (47.0)	168 (28.2)	**<0.0001**
Present CVD or risk factors	722 (81.9)	708 (61.4)	**<0.0001**	159 (83.3)	333 (59.7)	**<0.0001**	563 (81.5)	375 (62.9)	**<0.0001**
Income status, income < $731	127 (14.4)	762 (66.2)	**<0.0001**	38 (19.9)	407 (73.2)	**<0.0001**	89 (12.9)	355 (59.6)	**<0.0001**
Living status, with family	832 (94.3)	1,056 (91.8)	**0.03**	180 (94.2)	522 (94.1)	0.92	652 (94.4)	534 (89.8)	**0.002**
Awareness, unaware	100 (11.3)	172 (14.9)	**0.02**	30 (15.7)	121 (21.7)	0.08	70 (10.1)	51 (8.6)	0.33

*Statistical significance (P <0.05) was indicated in bold*.

## Results

The mean ± standard deviation age of the 2,036 participants with previous TIA was 64.8 ± 10.2 years, of which 39.7% were males. As shown in Table 1, 36.8% of participants with TIA still failed to seek EMS once stroke symptoms occur. A significant difference existed in the distribution of the factors between groups by intention to call EMS, including education, urban–rural residency, medical insurance type, awareness of stroke symptom, income, stroke history of families or friends, and presence of cardiovascular disease or risk factors. Stratified by urban–rural residency (Table 2), the proportion of not calling EMS was 48.4% among rural participants, significantly higher than that (21.7%) among urban participants. A significant urban–rural difference was observed in the distribution of the factors among all participants with previous TIA or the subsample by intention to call EMS.

**Figure 1:**
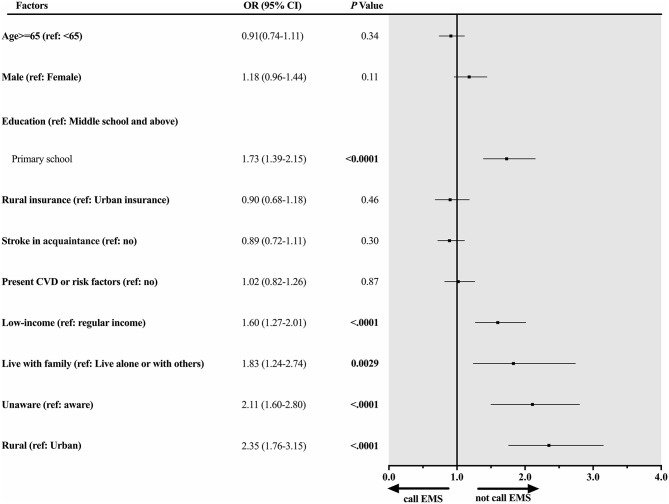
Factors associated with not calling EMS among all participants with previous TIA. Forest plot and corresponding statistical results (OR, 95% CI, and *p-*value) illustrate risk factors associated with not calling EMS among all participants with previous TIA.

In the multivariate regression analysis among all participants with previous TIA (Figure 1), factors significantly associated with not calling EMS were primary school education [odds ratio (OR) 1.73, 95% confidence interval (CI) 1.39–2.15], rural residency (OR 2.35, 95% CI 1.76–3.15), living with family (OR 1.83, 95% CI 1.24–2.74), unaware stroke symptoms (OR 2.11, 95% CI 1.60–2.80), and low income (OR 1.60, 95% CI 1.27–2.01).

**Figure 2:**
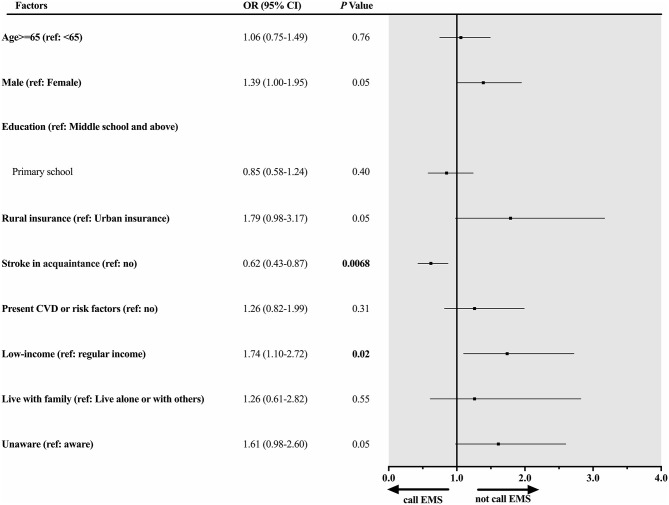
Factors associated with not calling EMS among urban participants with previous TIA. Forest plot and corresponding statistical results (OR, 95% CI, and *p-*value) illustrate risk factors associated with not calling EMS among urban participants with previous TIA.

**Figure 3:**
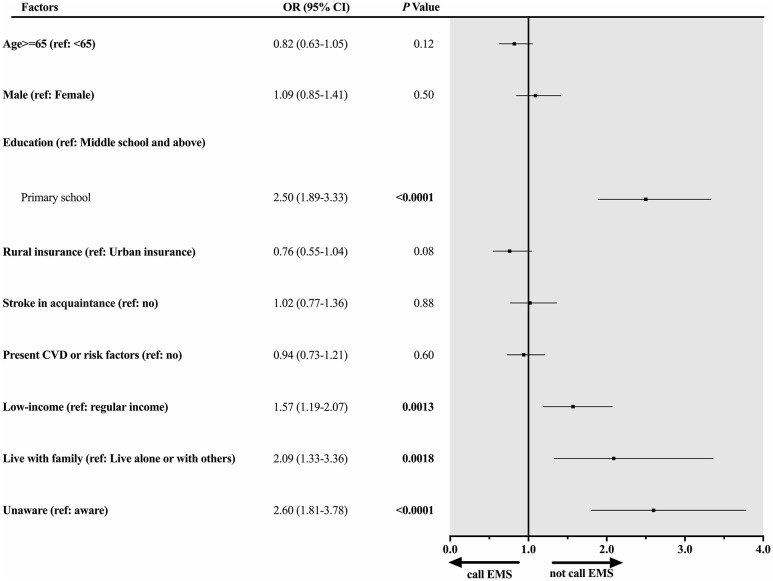
Factors associated with not calling EMS among rural participants with previous TIA. Forest plot and corresponding statistical results (OR, 95% CI, and *p-*value) illustrate risk factors associated with not calling EMS among rural participants with previous TIA.

Stratified by urban–rural residency, among urban participants (Figure 2), low income was significantly associated with an increased risk of not calling EMS (OR 1.74, 95% CI 1.10–2.72). However, stroke in acquaintance (family members or friends) was significantly associated with a decreased risk of not calling EMS (OR 0.62, 95% CI 0.43–0.87). Among rural participants (Figure 3), besides the low income (OR 1.57, 95% CI 1.19–2.07), additional risk factors were significantly associated with not calling EMS, including primary school education (OR 2.50, 95% CI 1.89–3.33), living with family (OR 2.09, 95% CI 1.33–3.36), and unaware stroke symptoms (OR 2.60, 95% CI 1.81–3.78).

## Discussion

Our study showed that 36.8% of participants with TIA still failed to seek EMS once stroke symptoms occur, which was only slightly lower than that in the general population (39.1%, *n* = 187,723 among all FAST-RIGHT study participants) (9). Rural participants with previous TIA had a higher percentage and risk of not calling EMS than in urban areas. Multiple risk factors among rural participants were identified, including primary school education, living with family, unaware stroke symptoms, and low-income. Nonetheless, among urban participants, low-income was the primary risk factor.

The findings suggested that the presence of previous TIA history does not significantly improve the intention of calling EMS compared with the general population. It is particularly remarkable among rural participants. Thus, priority in stroke-reduction strategy needs to be given to the rural areas. This also conforms to the fourth core principle of Health China 2030 (HC 2030)—fairness and justice. The country's rural areas are given special attention to promote equal access to basic public health services and maintain public welfare (25). Specifically, we need to formulate countermeasures that incorporate the multifaceted educational (low education level, unawareness of stroke symptoms), cultural (living with family), and financial (low-income) factors. Therefore, existing or newly developed stroke education programs need to expand their coverage and ensure efficient delivery of stroke knowledge to vulnerable high-risk people in rural areas. It is not surprising to find that living with family increased the likelihood of not calling EMS in rural areas. One explanation is that traditionally, the first reaction is to call family members instead of EMS when feeling uncomfortable among many rural residents, especially older people. It also may be due to a lack of relevant knowledge to distinguish how urgent the situation is, which needs to call EMS to save time. Another reason could be due to the lack of an efficient EMS system in remote rural areas. Thus, it is equally important to change the traditional way of thinking and action and enhance access to the EMS service in rural areas. The same challenge in urban and rural areas is how to improve the EMS rate in the low-income population. To solve this, reducing the percentage of the out-of-pocket payment in total health expenditure in the low-income population is crucial to alleviate the financial burden for medical care and eliminate the poverty caused by illness (26). However, how to avoid EMS resource abuse also needs deliberate strategy planning.

Factors associated with seeking EMS could be varied in different countries due to the differences in social culture and perceptions, health care system, and socioeconomic development. For example, the Oxford vascular study in the United Kingdom found that seeking first aid from their general practitioners was a significant factor for the prehospital delay, especially when the symptoms occurred during off-work hours (27). However, the correct recognition of symptoms was not associated with less delay in their study. By contrast, most Chinese people usually seek medical service from local hospitals that they trust but does not have their personal general practitioners. Therefore, a better understanding of the reasons why patients do not use the EMS in different populations will improve stroke care quality and reduce stroke incidence.

Our study's strengths are the representativeness in that the sample covered most provinces in China with carefully designed sampling and research methods (23, 24). It emphasized the importance of recognizing factors that hinder seeking EMS in urban and rural residents with previous TIA. Nevertheless, several potential limitations need to be considered. First, the TIA diagnosis was based on patients' self-report. Although the study physician further inquired or checked medical records for relevant symptoms, signs, and imaging results to confirm the diagnosis, information bias still likely existed due to the retrospective method. Second, the seeking of EMS was based on the participant's intention but not actual action; therefore, it may not reflect their real-world options. Third, this study is a cross-sectional design that revealed the co-existence and the association between risk factors and the intention of not calling EMS. However, it cannot indicate a direct causal relationship. Lastly, our study findings might only be transferrable to other countries with similar stroke management pathways.

In conclusion, rural residents with previous TIA in China had a higher percentage of not calling EMS and had multifaceted risk factors compared with urban residents. Our findings will contribute to formulating pertinent countermeasures to alleviate China's social and economic burden.

## Data Availability Statement

The raw data supporting the conclusions of this article will be made available by the authors, without undue reservation.

## Ethics Statement

The studies involving human participants were reviewed and approved by The Peking Union Medical College Hospital Institutional Review Board. The patients/participants provided their written informed consent to participate in this study.

## Author Contributions

S-DL, C-PG, and BP: data acquisition. JY and G-LS: statistical analysis. JY: manuscript preparation. G-LS and BP: manuscript editing. C-PG and L-YC: manuscript review. All authors: concept, design, and definition of intellectual content.

## Conflict of Interest

The authors declare that the research was conducted in the absence of any commercial or financial relationships that could be construed as a potential conflict of interest.
